# Supplementary material for: The nested structural organization of the worldwide trade multi-layer network
Source: Sci Rep. 2019 Feb 27;9:2866. doi: 10.1038/s41598-019-39340-w (PMC6393514; doi:10.1038/s41598-019-39340-w)
Supplement: Supplementary file 1 — Supplementary Information file [file 41598_2019_39340_MOESM1_ESM.pdf]

The nested structural organization of the worldwide trade multi-layer network

Luiz G. A. Alves[1,*], Giuseppe Mangioni[2], Isabella Cingolani[3], Francisco Aparecido Rodrigues[1,8,9], Pietro Panzarasa[4], and Yamir Moreno[5,6,7]

[1]Institute of Mathematics and Computer Science, University of São Paulo, São Carlos, SP 13566-590, Brazil
[2]Dipartimento di Ingegneria Elettrica, Elettronica e Informatica, University of Catania, Catania 95125, Italy
[3]Big Data and Analytical Unit, Department of Surgery and Cancer, Imperial College London, London SW7 2AZ, UK
[4]School of Business and Management, Queen Mary University of London, London E1 4NS, UK
[5]Department of Theoretical Physics, University of Zaragoza, Zaragoza 50009, Spain
[6]Institute for Biocomputation and Physics of Complex Systems, University of Zaragoza, Zaragoza 50009, Spain
[7]ISI Foundation, Torino 10126, Italy
[8]Mathematics Institute, University of Warwick, Gibbet Hill Road, Coventry CV4 7AL, UK
[9]Centre for Complexity Science, University of Warwick, Coventry CV4 7AL, UK
[*]lgaalves@usp.br
[+]All authors contributed equally to this work

Data description

Table S1 the 56 NACE Rev.2 economic activities divisions included in the WIOD, and Table S2 shows the list of 43 countries (excluding the Rest of the World).

NACE Rev. 2 Division	Economic activity description
A01	Crop and animal production, hunting and related service activities
A02	Forestry and logging
A03	Fishing and aquaculture
B	Mining and quarrying
C10-C12	Manufacture of food products, beverages and tobacco products
C13-C15	Manufacture of textiles, wearing apparel and leather products
C16	Manufacture of wood and of products of wood and cork, except furniture; manufacture of articles of straw and plaiting materials
C17	Manufacture of paper and paper products
C18<br>C19	Printing and reproduction of recorded media<br>Manufacture of coke and refined petroleum products
C20	Manufacture of chemicals and chemical products
C21	Manufacture of basic pharmaceutical products and pharmaceutical preparations
C22	Manufacture of rubber and plastic products
C23	Manufacture of other non-metallic mineral products
C24	Manufacture of basic metals
C25	Manufacture of fabricated metal products, except machinery and equipment
C26	Manufacture of computer, electronic and optical products
C27	Manufacture of electrical equipment
C28	Manufacture of machinery and equipment n.e.c.
C29	Manufacture of motor vehicles, trailers and semi-trailers
C30	Manufacture of other transport equipment
C31_C32	Manufacture of furniture; other manufacturing
C33	Repair and installation of machinery and equipment
D35	Electricity, gas, steam and air conditioning supply
E36	Water collection, treatment and supply
E37-E39	Sewerage; waste collection, treatment and disposal activities; materials recovery; remediation activities and other waste management services
F	Construction
G45	Wholesale and retail trade and repair of motor vehicles and motorcycles
G46	Wholesale trade, except of motor vehicles and motorcycles
G47	Retail trade, except of motor vehicles and motorcycles
H49	Land transport and transport via pipelines
H50	Water transport
H51	Air transport
H52	Warehousing and support activities for transportation
H53	Postal and courier activities
I	Accommodation and food service activities
J58	Publishing activities

Continued on next page

Table S1 – continued from previous page

NACE Rev. 2 Division	Economic activity description
J59_J60	Motion picture, video and television programme production, sound recording and music publishing activities; programming and broadcasting activities
J61	Telecommunications
J62_J63	Computer programming, consultancy and related activities; information service activities
K64	Financial service activities, except insurance and pension funding
K65	Insurance, reinsurance and pension funding, except compulsory social security
K66	Activities auxiliary to financial services and insurance activities
L68	Real estate activities
M69_M70	Legal and accounting activities; activities of head offices; management consultancy activities
M71	Architectural and engineering activities; technical testing and analysis
M72	Scientific research and development
M73	Advertising and market research
M74_M75	Other professional, scientific and technical activities; veterinary activities
N	Administrative and support service activities
O84	Public administration and defense; compulsory social security
P85	Education
Q	Human health and social work activities
R_S	Other service activities
T	Activities of households as employers; undifferentiated goods- and services-producing activities of households for own use
U	Activities of extraterritorial organizations and bodies

Table S1. Economic activities in WIOD 2016 Release

Country name (ISO Alpha-3 Code)
Australia (AUS), Austria (AUT), Belgium (BEL), Bulgaria (BGR), Brazil (BRA), Canada (CAN), Switzerland (CHE), China (CHN), Cyprus (CYP), Czech Republic (CZE), Germany (DEU), Denmark (DNK), Spain (ESP), Estonia (EST), Finland (FIN), France (FRA), United Kingdom (GBR), Greece (GRC), Croatia (HRV), Hungary (HUN), Indonesia (IDN), India (IND), Ireland (IRL), Italy (ITA), Japan (JPN), Korea, Rep. (KOR), Lithuania (LTU), Luxembourg (LUX), Latvia (LVA), Mexico (MEX), Malta (MLT), Netherlands (NLD), Norway (NOR), Poland (POL), Portugal (PRT), Romania (ROU), Russian Federation (RUS), Slovak Republic (SVK), Slovenia (SVN), Sweden (SWE), Turkey (TUR), Taiwan (TWN), United States (USA)

Table S2. Countries in WIOD 2016 Release

Supplementary figures

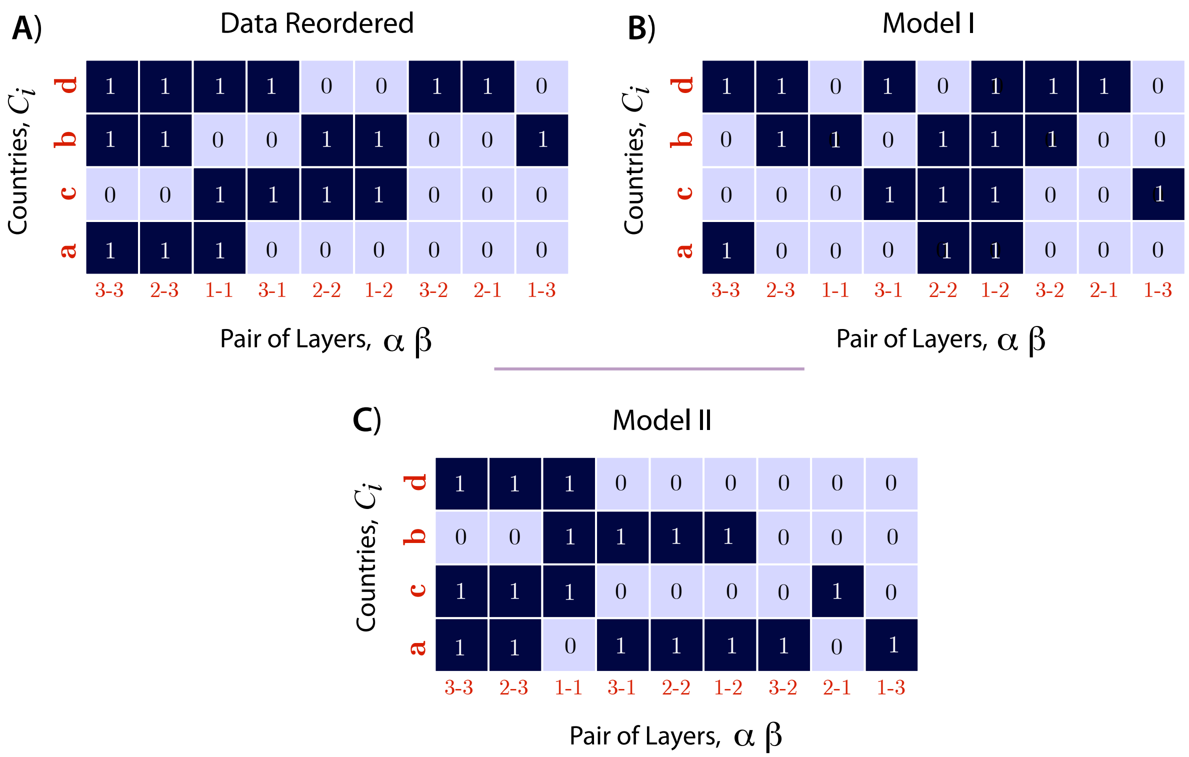

Figure S1. A simple example of the two null models for testing sellers' country-based and transaction-based nestedness. A) The original participation matrix in which the rows are four exporters and the columns are nine different combinations of three layers (industries). The matrix has been reordered by row/column degree (i.e., number of ones). B) *Model I*. For each row at a time, columns are reshuffled within (three) blocks defined by the common exporting layer (i.e., block [1-1, 1-2, 1-3], block [2-1, 2-2-, 2-3], and block [3-1, 3-2, 3-3]). Within each block, the ones are randomly reshuffled, while the total number of ones is preserved. For example, in block [1-1, 1-2, 1-3] the one moves from column [1-1] in the original data to column [1-2] in the null model. Notice that this model randomly reassigns end layers to the starting layers of transactions while keeping the out-degree of each country unchanged both within each layer and across the whole network. C) *Model II*. For each layer at a time, rows are reshuffled by (three) blocks of columns defined by the common exporting layer. Within each block, the entire rows are randomly swapped, while the total number of ones is preserved. For example, in block [1-1, 1-2, 1-3] the entries of row *b* have been swapped with the entries of row *a*. Notice that this model randomly reassigns countries to transactions starting at a given layer while keeping the degree distribution of layers across the whole network. Moreover, the out-degree distribution of countries is preserved within each layer, but not across the whole network.

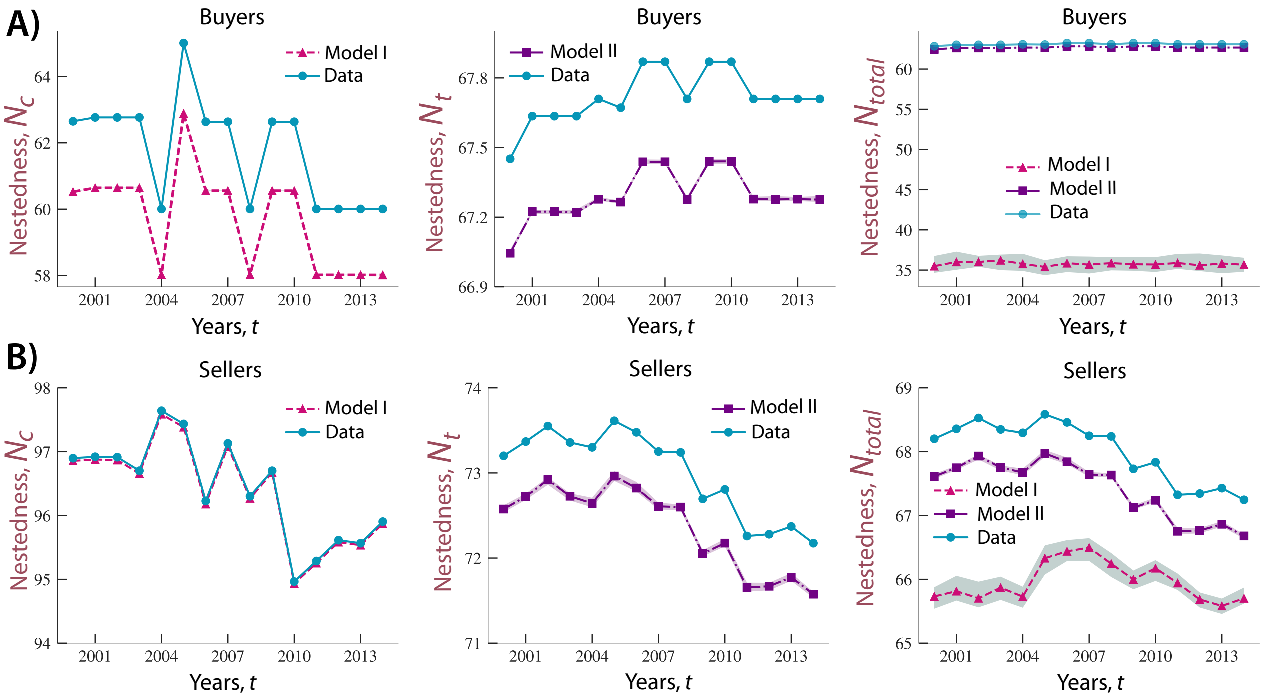

Figure S2. Null models compared with real data. Buyers' (A) and sellers' (B) nestedness compared with values obtained with the null models. The nestedness of countries is always higher than the values obtained by using *Model I*, that is by using a multi-layer network in which connections are randomly reshuffled but that preserves the same node degree distribution (i.e., the global and intra-layer degree distributions) as in the real network. The nestedness of production stages (transactions) is also higher than the one found using *Model II*, that is by using a multi-layer network in which connections are randomly reshuffled among the countries in the same layer, but that preserves the countries' intra-layer degree distribution (in-degree distribution for buyers and out-degree distribution for sellers) for each layer as well as the layers' degree distribution. All observed values of country-based, transaction-based, and total nestedness are outside the 95% confidence interval of nestedness obtained using the corresponding null models over 1,000 replicates. Notice that the thin shaded areas reflecting confidence intervals are explained by the small variation in values obtained using null models based on very dense participation matrices (i.e., percentage of ones in the matrix is $\approx 90\%$).

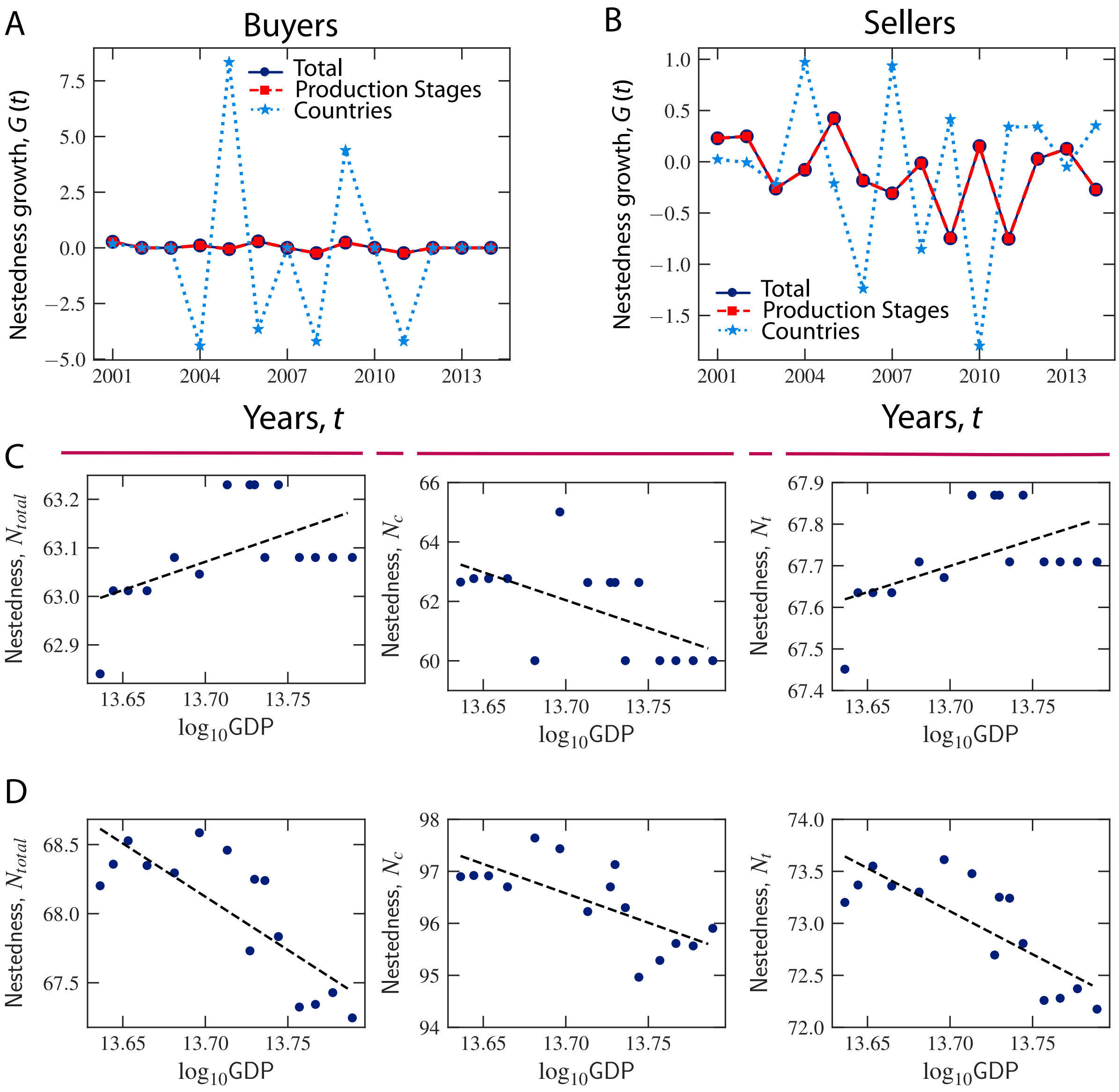

Figure S3. Variations in nestedness and relationship between nestedness and GDP. A) Growth in global nestedness calculated over the years from 2000 to 2014 from the buyers' perspective. B) Growth in global nestedness calculated over the years from 2000 to 2014 from the sellers' perspective. C) Relationship between buyers' nestedness and total GDP. D) Relationship between sellers' nestedness and total GDP. The black dashed lines are the OLS fit to Eq. 7 (see main text). See Table 1 in the main text for the estimated regression coefficients.
